# Application of Polymeric Tubular Ultrafiltration Membranes for Separation of Car Wash Wastewater

**DOI:** 10.3390/membranes14100210

**Published:** 2024-09-28

**Authors:** Piotr Woźniak, Marek Gryta

**Affiliations:** Faculty of Chemical Technology and Engineering, West Pomeranian University of Technology in Szczecin, ul. Pułaskiego 10, 70-322 Szczecin, Poland; piotr.wozniak@zut.edu.pl

**Keywords:** ultrafiltration, tubular membrane, car wash wastewater, fouling, membrane washing

## Abstract

The commercial ultrafiltration tubular polyvinylidene fluoride (PVDF) (100 and 200 kDa) and polyethersulfone (PES) (4 kDa) membranes were applied for filtration of car wash wastewater. Intensive fouling was noticed, which caused an over 50% flux reduction during 3–5 h of the filtration process. This phenomenon was reduced by washing the membranes with an alkaline cleaning agent (pH = 11.5), which is used in car washes to remove insects. The filtration/membrane washing cycle was repeated many times to achieve stable operation of the membrane modules. It has been found that cyclic repeated washing did not deteriorate the performance of the membranes. Despite frequent cleaning of the membranes (every 5–7 h), irreversible fouling occurred, resulting in a 20% reduction in the initial permeate flux. However, the formation of a filter cake definitely improved the separation degree and, for the 200 kDa membranes, separation of the wastewater components was obtained as it was for the 4 kDa membranes, while, at the same time, the permeate flux was 5 times higher.

## 1. Introduction

Vehicle washing services are provided by various manual and automatic car washes [1,2,3]. These facilities generate many problems, such as water contamination and requirements for its purification. Their magnitude largely depends on the type of car washing technologies being used. In order to protect the environment, in many countries, cars can only be washed in a commercial car wash, where wastewater has to be pre-treated before being discharged into the municipal wastewater treatment system. The most common are automatic car washes, mostly placed at gas stations or supermarkets, which use high-pressure washing and brush or soft cloth washing operations [1,2]. In this case, additional treatment systems are often used to allow re-use of the wash water. Nowadays, several physical methods are used to treat car wash wastewater such as gravity separation, skimming, and filtration, and chemical methods like coagulation, flocculation, electro-coagulation, advanced oxidation, membrane separation, and biological treatment [3,4,5,6,7,8].

The use of brushes to wash cars can damage the paintwork; hence, the rapid development of touchless technologies in recent years has been observed, and these technologies are gaining popularity for washing passenger cars. There are such car washes in almost every city, and there are several thousand in Poland [2]. These are self-service car washes, where the customer can choose and apply any programme and washing time. Possible options include rinse water, active foam, insect removal, wheel cleaning, rinsing with osmotic water, and waxing for paint protection. Finally, the car is rinsed with spot-free water (desalinated by reverse osmosis) which allows for an air-dry application. Washing water and cleaning agents are sprayed onto the car using the high-pressure nozzles (wash pipes or spray guns) [1,2]. The liquid is discharged under high pressure (10 MPa), which requires caution as there is a known case of pressurised water spray having damaged the user’s foot [9].

The touchless washes usually have 2–3 of the washing stand paving connected to the wastewater treatment system. Such stations only allow up to 100 cars to be washed per day. This limits the financial returns; hence, the expensive wastewater treatment systems that are installed at large automatic stations washing several thousand cars per day are not applicable here [2,10]. For this reason, at touchless car washes, the created wastewater is collected in a settling tank (removal of coarse particles and sand) from which it flows through an oil separator into sewage systems. In such an arrangement in Poland, the charge for 1 m^3^ of tap water with treatment is about 5–7 Euro. Such low costs discourage car wash owners from investing in wastewater treatment and re-use of water. However, progress in reusing the wastewater by setting up rules and strict regulations is increasing interest in implementing cheap and simple methods of recovering at least part of the used wash water.

At minimum, the wash water produced from wastewater must be free of at suspended particles, oil, grease, and microorganisms. Such effects can be achieved using the ultrafiltration (UF) process [3,11,12,13,14]. Spiral-wound modules are popularly used in industrial UF installations. However, their use may not be feasible when the effluent has a high turbidity, resulting in rapid clogging of the mesh-filled channels in such modules [15]. For this reason, to reduce fouling, it is proposed to use pre-treated feed water [3,11,12]. However, such a solution complicates and increases the installation cost. Therefore, in the technology studied for the touchless washer, the feed water is taken directly from the settling tank. In this case, the problem of high turbidity can be solved by using tubular membranes that allow the separation of solutions containing suspended solids [16,17,18].

Agents applied for car washing contain detergents and often alkalis (e.g., NaOH), which can cause membrane degradation [15,19]. In addition, the ingredients in the wastewater cause significant fouling, so chemical cleaning of the membranes is necessary [8,20]. For these reasons, UF installations should be equipped with membranes with high chemical resistance. Such properties are found in ceramic membranes, which show high resistance to fouling and cleaning agents [17]; however, they are a more expensive solution than polymeric membranes [12]. Moreover, the separation of certain types of feed necessitates the use of multi-stage chemical cleaning programmes for ceramic membranes [21], which increases costs. This can restrict the application of ceramic membranes in small car washes.

High chemical resistance has been demonstrated by hydrophobic polymers such as polyethersulfone (PES) and polyvinylidene fluoride (PVDF), which are often used for UF membranes [22,23,24,25]. Hydrophobic membranes show a higher susceptibility to fouling, so manufacturers often add wetting agents such as polyvinylpyrrolidone (PVP) or polyvinyl alcohol [25]. The disadvantage is that the chemical resistance of such modified membranes is reduced [26,27]. Moreover, new types of membranes are being intensively investigated for the treatment of oily wastewaters [28,29]. Two-dimensional (2D) materials have been utilised to develop membranes, which can demonstrate promising oil–water separation efficiency and flux [28]. However, for the industrial implementation of such membranes, in addition to starting their production, it is necessary to demonstrate their long-term durability. For this reason, this study investigated the resistance of applied industrial tubular membranes to car wash wastewater.

Membrane fouling is indicated as a serious obstacle that limits the application of UF [11,12,30]. Nevertheless, publications showing car wash wastewater separation and UF membrane washing effects are scarce. There are no papers presenting changes in membrane performance during long-term separation of real car wash wastewater with cyclic repetition of chemical membrane cleaning. Such information is necessary to be able to implement the UF process for car wash water treatment. In the work presented, alkaline cleaning agents (pH > 11) being used at car washes were also used to wash the membranes. This solution eliminates the purchase of additional cleaning agents and ensures the use of agents approved for car washing. On the other hand, the effectiveness of washing the membranes with this method is not known. Therefore, the effect of separated wastewater and alkaline cleaning agents on the performance of the applied tubular membranes was investigated in tests lasting several weeks.

## 2. Materials and Methods

### 2.1. UF Procedure

**Figure 1:**
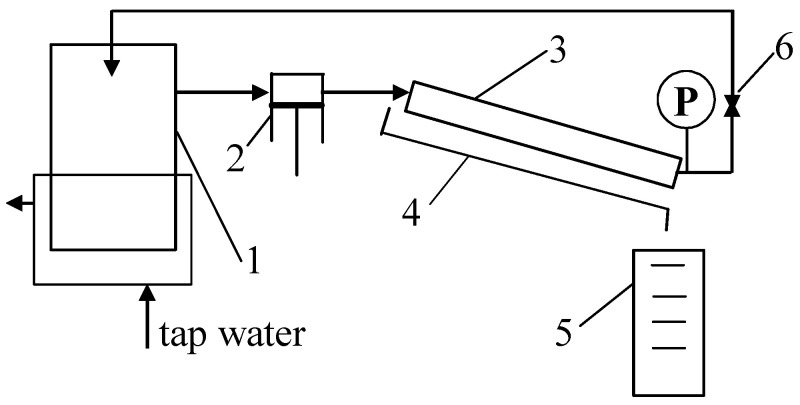
UF experimental set-up. 1—feed tank, 2—pump, 3—tubular membrane, 4—permeate collector, 5—measurement cylinder, 6—valve, and P—manometer.

Ultrafiltration tubular membranes with a diameter of 12.5 mm manufactured by PCI [31] were used to conduct the study. The membranes used were FP100 and FP200 (PVDF), as well as ESP04 membranes made of modified PES. The parameters of the membranes are shown in Table 1. Membrane samples with a length of 25 cm were installed in the system, as schematically shown in Figure 1.

The investigations of the UF process were carried out in a cross-flow mode. The feed flowed inside the membrane at a speed of 1 m/s. Transmembrane pressure (TMP) was equal to 0.1 MPa. The feed tank was cooled using tap water, and the feed temperature was 293–295 K. Wastewater separation studies were conducted in batch mode, using two methods of permeate collection. In the first method, the permeate was recycled to a feed tank (VF = 3 L) to maintain a constant concentration of the feed. In the second mode, the permeate was collected from installation; thus, the feed concentration was gradually increased during the UF process. In this case, the water recovery coefficient can be expressed by volume concentration ratio (VCR), which was calculated with the following equation:

(1) $$\mathrm{VCR}=\frac{V_{F}}{V_{F}-V_{P}}$$

where V_F_ and V_P_ are volume of the feed and permeate, respectively.

Wastewater separation was carried out in two stages. In the first stage, 1.5 L of permeate (VCR = 2) was obtained from the feed (3 L). The 5–6 wastewater samples were separated. The resulting retentates were mixed together and separated in a second stage, again halving the volume of the feed (total VCR = 4).

Synthetic and real car wash wastewater were used for the UF tests. During their separation, a decrease in the permeate flux occurred due to fouling. Therefore, the membranes were washed cyclically with alkaline cleaning agents. The cleaning efficiency was determined by measuring the pure water flux (feed—deionised (DI) water) after each membrane cleaning. During the UF process, the separation properties of the membranes can change due to fouling and cyclic chemical cleaning. To determine these properties, changes in the degree of dextran separation, chemical oxygen demand (COD), and surfactants were studied.

### 2.2. Solutions Composition

Real wastewater taken from the settling tank at touchless car washes was used for the UF testing. The parameters of these wastewaters are presented in Table 2. At these car washes, a foaming agent (Turbo Foam–EuroEcol, Łódź, Poland) and a polymeric wax solution for paint maintenance (Hydrowax-EuroEcol) were used to wash the cars. These agents contained anionic and nonionic surfactants, and their composition is presented in [15]. A mixture of these agents (0.5% Turbo Foam + 0.2% Hydrowax) as synthetic wastewater was also used to test the UF process.

As it has been indicated previously, during the UF process, a decrease in the permeate flux is due to the fouling phenomenon. For this reason, the membranes were washed cyclically using alkaline cleaning agents. For cleaning the membranes tested, the PCI manufacturer recommends P3 Ultrasil 11 solutions (pH = 12) [32,33]. In this study work, the membranes were washed with 0.5% Insect solution (pH = 11.5) used at the car wash to remove insects from cars. Insect solution, like P3 Ultrasil 11, contains NaOH and detergents. In previous works, this Insect solution was shown to effectively clean PES membranes contaminated by car wash wastewater [19]. Membrane washing is often carried out with hot solutions (323–333 K), which increases costs. Therefore, in this study the washing process was carried out without heating, and solutions were used at ambient temperature (about 293 K).

New membranes are factory preserved; thus, to remove preservatives prior to testing, the membranes were washed with Insect solution (1 h) and an initial permeate flux (TMP = 0.1 MPa) was determined after rinsing with DI water. Membranes prepared in this way were used for further UF tests.

Dextran solutions (Polfa, Warszawa, Poland) with molecular weight cut-offs (MWCO) in the range 10–1000 kDa and a concentration of 0.5 g/L were used to study changes of membrane separation properties.

### 2.3. Analytical Methods

The Hach cuvette tests (Hach Lange, Wrocław, Poland) were used to determine the concentration of surfactants (LCK 334-nonionic, LCK 344-anionic) and COD (LCK 1014). The biological oxygen demand (BOD) was determined by using test LCK 555, and the total P and total N by using LCK 348 and LCK 238 tests, respectively.

The concentration of dextrans was analysed using a high-performance liquid chromatograph (UlitiMate 3000, Dionex, Sunnyvale, CA, USA) with a PolySep-GFC-P 4000 column (Phenomenex, Torrance, CA, USA).

The pH of each solution was measured using a 6P Ultrameter (Myron L Company, Carlsbad, CA, USA). The turbidity of the tested solutions was measured with a portable turbidity meter model 2100 AN IS with a detection limit of 0.01 NTU (Hach Company, Loveland, CO, USA).

The membrane morphology and deposit composition were studied using an SU8020 (Hitachi High Technologies Co., Tokyo, Japan) scanning electron microscope (SEM) coupled with energy dispersion spectrometry (EDS). All samples were sputter-coated with chromium. Membrane samples were obtained by cutting off about 2 cm of tubular membranes (inlet side), which made it possible to analyse the changes occurring on their surface during the various stages of the UF process.

## 3. Results

### 3.1. Membrane Performance

**Figure 2:**
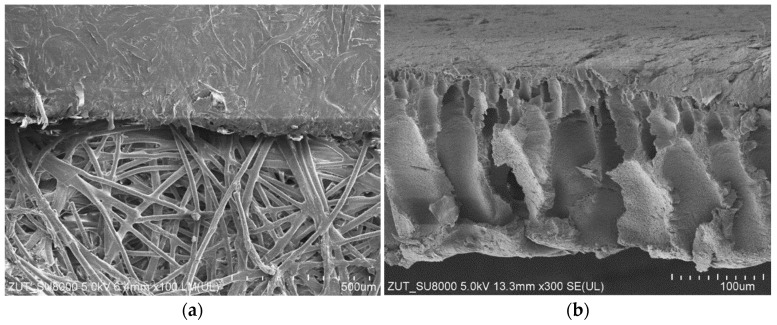
SEM images of tubular UF membranes. (**a**) ESP04 membrane (**top**) formed on the porous support layer (**down**) and (**b**) FP100 membrane cross-section; support layer was removed.

For the UF studies, composite tubular membranes were used, whose mechanical strength is provided by a non-woven support layer rolled up in the form of a 12.5 mm diameter tube. A polymer membrane is formed on its inner surface (Figure 2a). The membranes tested had a similar structure, which consisted of two layers. The first one had a thickness of about 150–200 μm and large finger-shaped pores. The second one, an external separation layer (skin layer), was less than 1 μm thick (Figure 2b); the size of the pores in this layer determines the separation degree.

**Figure 3:**
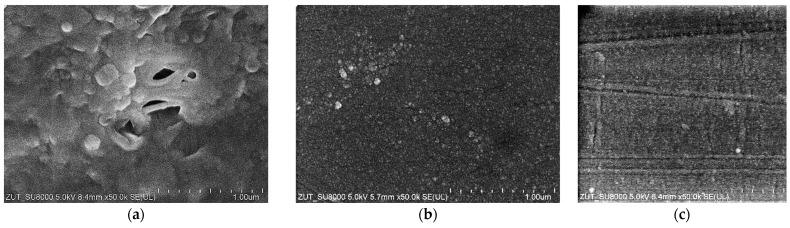
SEM images of membrane surfaces: (**a**) FP100, (**b**) FP200, and (**c**) ESP04.

The images of the membrane surfaces are shown in Figure 3. It can be seen that the membranes used differed significantly in their surface morphology. In the case of the FP100 membranes (Figure 3a), single pores with a size of less than 0.2–0.3 μm were found in some places. The most compact structure was that of the ESP04 membrane (Figure 3c), for which the declared MWCO value was only 4 kDa.

**Figure 4:**
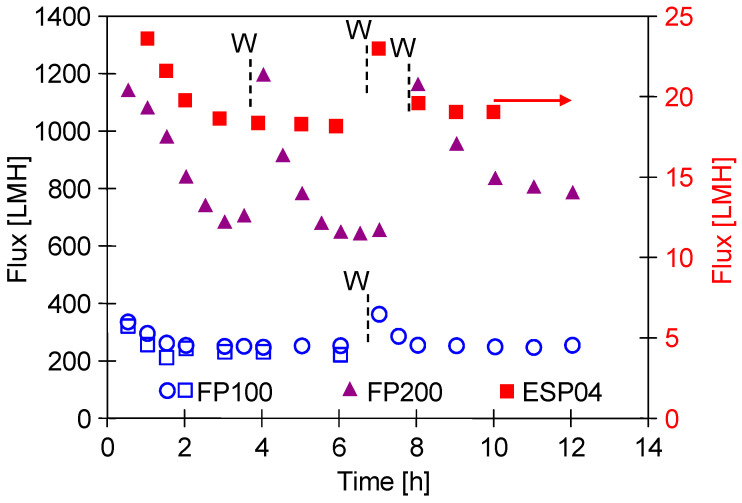
Changes in the initial permeate flux during filtration of deionised water. W—membranes washed with 0.5% Insect solution (30 min). TMP = 0.1 MPa.

It is well known that the permeate flux obtained for UF membranes depends on the permeability (porosity) of the external separation layer. The initial performance of pristine membranes obtained for DI water is shown in Figure 4. In each case, an increase in the performance was observed after washing the membranes with the use of Insect solution (PH = 11.5). This cleaning agent contains NaOH (3–5%). Consequently, it leads to changes in the polymeric structure of the membranes and increases their permeability [19,27]. As expected, the highest flux of approximately 1200 LMH was obtained for the FP200 membrane (200 kDa), while it was only 25 LMH for the ESP04 membrane (4 kDa). During the UF process, due to the compression and stabilisation of the polymeric membrane matrix, the flux obtained is usually much lower. For the membranes tested, it stabilised at 850 LMH (FP200), 240 LMH (FP100), and 18 LMH (ESP04). Similar significant changes in the permeate flux during the initial period of the UF membrane testing are presented in [34]. FP100 membranes from production periods differing by one year were tested; however, this did not affect their separation properties (Figure 4, symbol circle and open rectangular).

**Figure A1:**
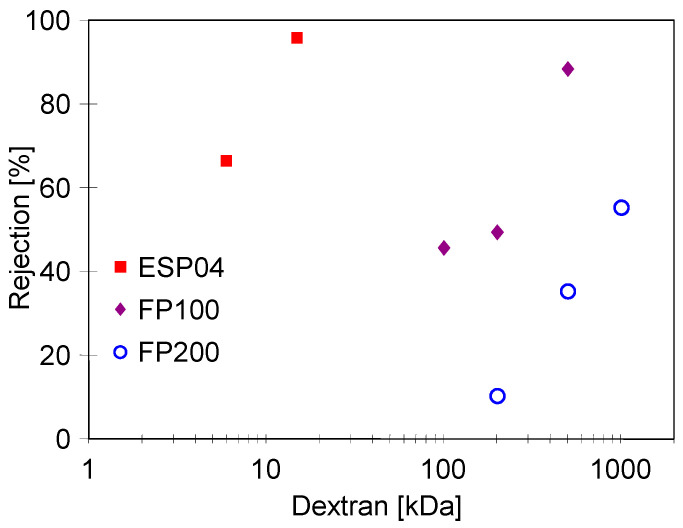
Dextran (0.5 g/L) rejection by studied pristine membranes pre-washed with the 0.5% Insect solution. TMP = 0.1 MPa.

After stabilising the permeate flux, the dextran separation tests were carried out (Appendix B, Figure A1). The values obtained differed significantly from the MWCO declared by the manufacturer. Similar discrepancies for pristine membranes were also found in other works [35].

### 3.2. UF Separation of Synthetic Wastewaters

It has been documented that membrane fouling can be caused not only by the contaminants removed from the cars but also by the cleaning agents used for membrane washing [15]. These deposits can be removed from the membrane surface with alkaline solutions, such as the Insect solution used at car washes (pH = 11.5). However, its cyclic and prolonged use causes slight damage to polyethersulfone membranes [19]. Such damage deteriorates the separation properties of the membranes. Therefore, in the present study, in a first step of experimental investigations, synthetic wastewater (Turbo Foam + Hydrowax) was used to test the resistance of the tubular membranes used.

**Figure 5:**
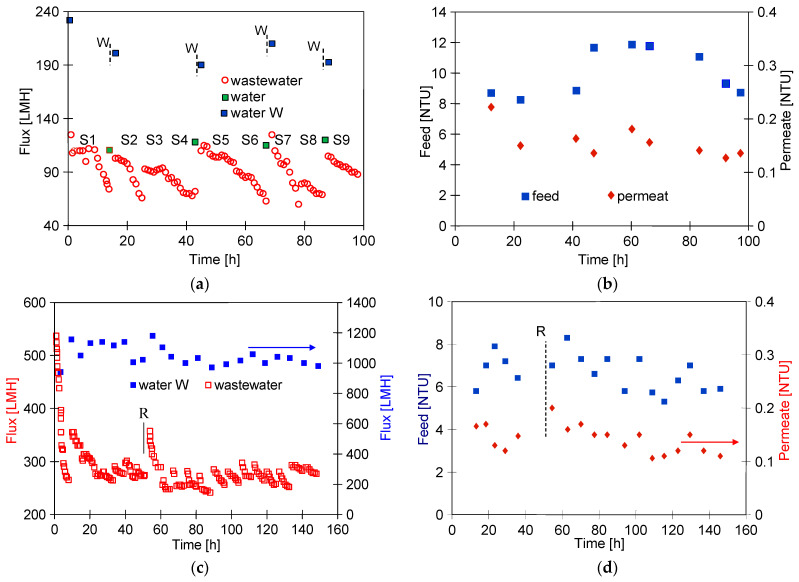
Changes in the permeate flux and turbidity of the feed and permeate during separation of the Turbo Foam + Hydrowax mixture. Membranes: (**a**,**b**) FP100 and (**c**,**d**) FP200. Water and water W—pure water flux (DI water as a feed) measured after the UF process and after washing the membrane with 0.5% Insect solution (30 min), respectively. Point R—rinse time 2 h.

**Figure 6:**
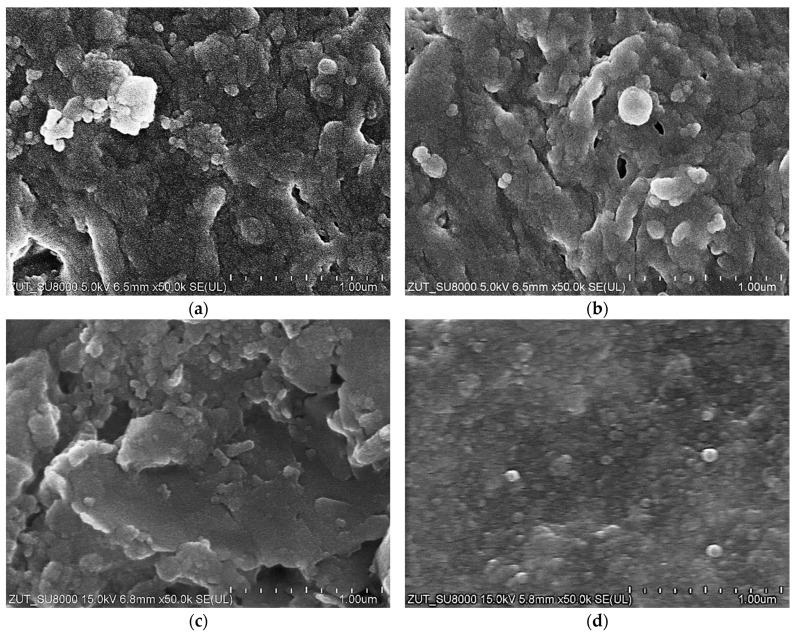
SEM images of the FP100 and FP200 membrane surfaces. Deposits formed during separation of the Turbo Foam + Hydrowax mixture: (**a**) FP100 and (**c**) FP200. Deposits removed by washing with Insect solution: (**b**) FP100 and (**d**) FP200.

Ongoing UF tests of the detergent–hydrowax mixture confirmed a systematic decrease in the permeate flux, similar to that observed for flat PES membranes [15]. For the FP100 membrane, the permeate flux during the mixture separation decreased below 70 LMH in each series (Figure 5a). Measurement series (S1–S9) were run 5–6 h/day, after which the feed was left overnight in the module. Periodically, every 5 days, the module was rinsed with Insect solution, which increased the permeate flux to a value close to the initial level. However, the pure water flux determined after washing the membranes 4 times decreased from 235 to 190 LMH (Figure 5a, W—90 h), indicating the occurrence of irreversible fouling. SEM studies confirmed that the washing applied did not remove all deposits from the membrane surface (Figure 6b). During the UF process, the turbidity of the permeate decreased from 0.21 to 0.13 NTU, indicating that the formation of a cake layer improved the separation (Figure 5b).

Similar results were obtained during synthetic wastewater separation using FP200 membranes. In this case, the membrane was washed after each UF run of 5–6 h, and, overnight, the system was filled with DI water, which additionally gave osmotic rinsing [36]. After the first 10 h of the UF process, the permeate flux decreased from 550 to 250 LMH (Figure 5c). As a result of repeated filtration/washing operations, changes in the permeate flux after 30 h of UF stabilised in the range of 250–300 LHM. SEM studies confirmed that the reduction in the flux was due to the fouling phenomenon (Figure 6c). Extension of washing time with the use of Insect solution from 0.5 to 2 h (Figure 5c, point R) increased the permeate flux to 560 LMH, but it also gave a slight increase in the permeate turbidity to 0.2 NTU (Figure 5d, R). These increases were due to the fact that most of the deposit was removed from the membrane surface (Figure 6d). However, after the separation of the next wastewater sample, the performance stabilised at the previous level of 250 LMH, and the turbidity was reduced to 0.1 NTU. It should be noted that, although the FP200 membranes retained the dextrans less well (Figure A1), as a result of the fouling, the turbidity of the permeate obtained (Figure 5d) was similar to that obtained for the FP100 membranes (Figure 5b).

**Figure 7:**
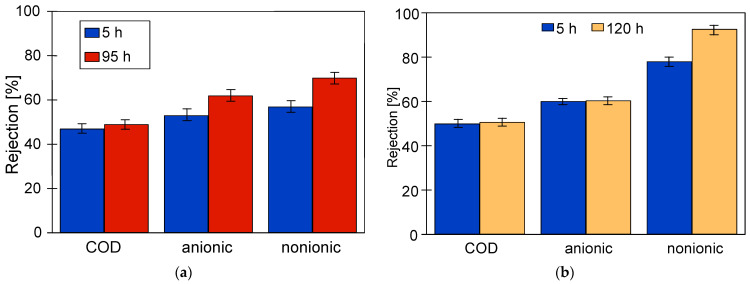
Changes in the rejection rate of COD and surfactants in samples taken at the beginning (5 h) and end (95 or 120 h) of the synthetic wastewater separation period. Membranes: (**a**) FP100 and (**b**) FP200.

The studies of permeate composition showed that the filter cake formed on the membranes improved not only the retention of suspended solids but also the separation of other wastewater components (Figure 7). Improved separation due to fouling was also presented in other works [37,38].

**Figure A2:**
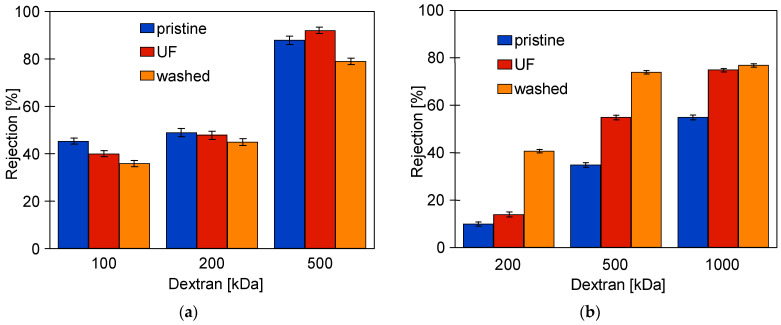
Changes in dextran separation FP100 (**a**) and FP200 (**b**) membranes. UF—membrane washed after UF process (30 min); washed—cleaning time 120 min.

SEM examination of the FP100-washed membrane surface showed the appearance of minor damage to the separation layer, in which pores above 0.1 μm were observed (Figure 6b). There were significantly more such pores than in the case of pristine membranes (Figure 3a), indicating that they were formed as a result of chemical membrane cleaning. Dextran separation tests for such a washed membrane confirmed that the presence of pores slightly worsened the separation (Figure A2). No such damage was found in the FP200 membrane (Figure 6d) and, after the UF testing of the wastewater, dextran separation was found to be improved (Figure A2b). Presumably, during the membrane washing, the finer deposits penetrated the pores, especially the largest ones. This had the effect of improving the separation properties of the skin layer, which could explain the improved retention of dextrans after the chemical cleaning of the FP200 membranes (Figure A2b, washed).

### 3.3. Ultrafiltration of Real Wastewater

In the next step, wastewater collected from the touchless car washes was used as a feed (Table 2). In the study, the separation of the wastewater and the obtained retentate was repeated several times. Examples of the compositions of the feed and permeate are given in Table A1. The permeate composition depended on the composition of the wastewater and the type of membranes used. The membranes tested retained components that affect the values of the parameters shown in the table, such as COD and BOD. As a result, the retentate contained significantly more suspension than the wastewater filtered in the first stage. Despite this, the composition of the permeate obtained in the second stage during filtration of the retentate (Permeate R) was similar to that of the permeates obtained from the new sample of wastewater. This shows that, despite the concentration of the feed, the composition of the permeate obtained changed slightly.

**Figure 8:**
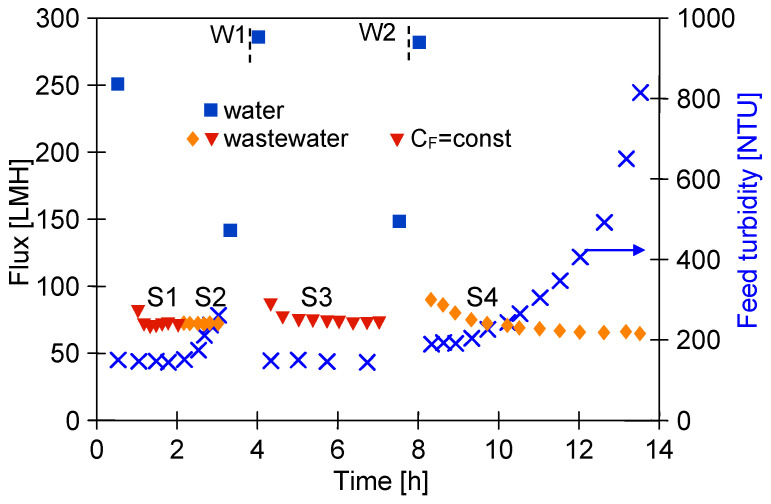
Separation of real wastewater (WW1) using FP100 membrane. Series: S1, S3—VCR = 1, S2–VCR = 2, and S4—VCR = 4. W1, W2—membrane rinsed with 0.5% Insect solution (30 min).

The results obtained during the UF process of WW1 wastewater using FP100 membranes are shown in Figure 8. During the filtration process with permeate return to the feed (C_F_ = const), the flux decreased to 70 LMH (series S1). After 2 h of the UF process, concentration of the feed was initiated, resulting in an increase in turbidity from 150 to 257 NTU (series S2). The turbidity of the permeate was 0.18–0.21 NTU, similar to the separation of synthetic wastewaters (Figure 5b). Despite the increase in concentration of the suspended solids, the permeate flux did not decrease and was still 70 LHM. After rinsing the installation with DI water, the pure permeate flux was 148.9 LHM and increased to 295.8 LHM after 30 min of washing with 0.5% Insect solution (Figure 8, point W1). Similarly, after separation of the next portion of the WW1 (series S3—C_F_ = const, 148 NTU), the pure water flux was 147.5 LHM and increased to 292.6 LHM after 30 min of washing with the Insect solution (W2). This result indicates that the twofold increase in concentration of suspended solids during the S2 series had little effect on the intensity of fouling. In the next series (S4), the filtrate was concentrated 4 times, resulting in an increase in turbidity to 810 NTU. The impact of the increasing concentration of suspended solids was greatest during the initial filtration period, during which, the filtration cake is formed. Further, the permeate flux stabilised at 65 LMH, but the increase in the fouling layer with increasing concentration is evidenced by improving separation since the permeate turbidity decreased from 0.35 to 0.23 NTU.

**Figure 9:**
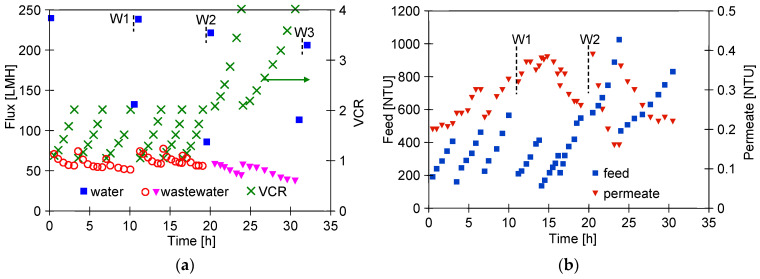
Course of WW2 wastewater separation by FP100 membranes: (**a**) flux and (**b**) feed and permeate turbidity. W1, W2, W3—membrane washed using 0.5% Insect solution (30 min). The last two series—retentates from previous runs (VCR = 2) used as a feed.

Another sample of FP100 membranes was used to separate the WW2 wastewater, during which, the permeate flux decreased to 50 LHM (Figure 9). This effluent contained fewer suspended solids than WW1, but likely more dispersed as indicated by its higher turbidity (190 NTU, Table 2). As a result, such a suspension may have formed a more compact filter cake, resulting in a greater decrease in flux than during WW1 separation. The presence of a finer suspension is also confirmed by the increase in turbidity of the permeate during the initial test period (Figure 9b) During the first 20 h of UF, six portions of the WW2 wastewater were filtered, reducing the feed volume by half (VCR = 2). It was observed that replacing the retentate with a new sample of WW2 cleaned the membranes, which may have facilitated the transport of suspended solids through the large pores in the membranes.

**Figure 10:**
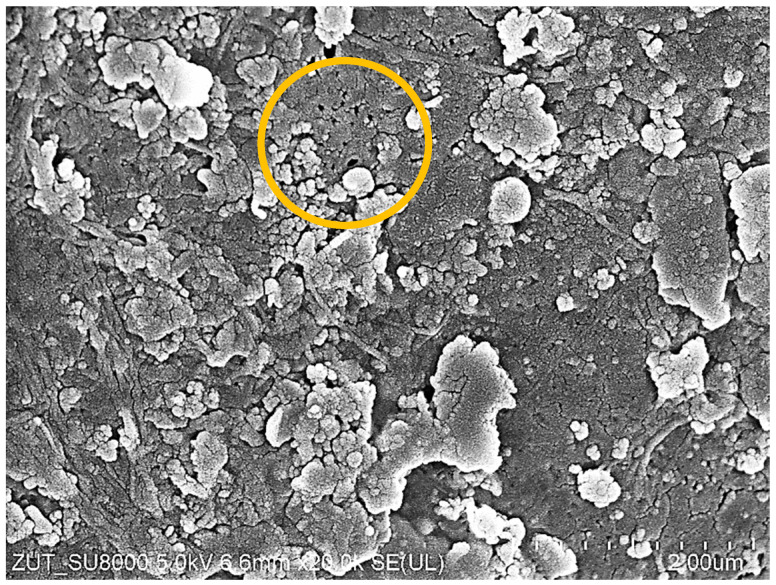
SEM images of FP100 membrane after UF wastewater WW2 and 30 min washing with 0.5% Insect solution (Figure 9, W1). Circle–area with pores.

A tubular module B1 cartridge (PCI) containing 18 pieces of FP100 membranes was purchased for the study. SEM examination of five membrane pieces showed that on the surface of some of the samples were pores as large as those shown in Figure 3a. It is likely that the membrane sample used for the WW2 effluent separation may have contained more of the pores, facilitating the permeation of the suspended solids into the permeate. The presence of such pores was found in the sample taken after the W1 wash (Figure 10). In the following hours of the UF process, there was stabilisation, and the turbidity of the permeate decreased with the time of the UF run, similar to the other cases studied. Re-washing (Figure 9b, W2) removed sediment from the membranes, which caused the permeate turbidity to increase again from 0.25 to 0.4 NTU; however, as the UF (filter cake recovery) process proceeded, turbidity decreased to 0.15 NTU. These membrane cleaning effects were also observed after replacing the retentate with a new portion of the effluent (Figure 9b). The results indicate that fouling and membrane washing stabilise the separation properties. This confirms the conclusions of another paper that membrane performance should only be assessed after several filtration/washing cycles [27,38].

**Figure 11:**
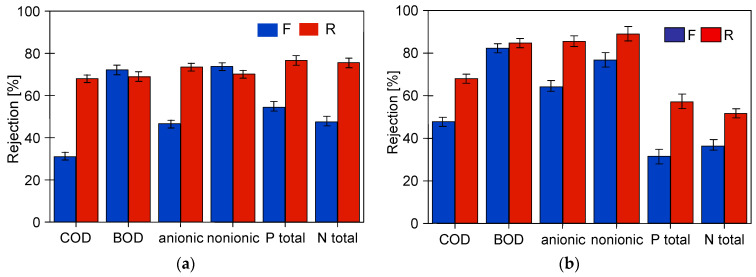
Variation in separation rates of FP100 membranes (**a**) WW1 and (**b**) WW2. F—filtration of the first batch of wastewater by pristine membranes. R—filtration of retentates (VCR = 4) in the last batch (13 h) presented in Figure 8 and 29 h in Figure 9a.

This conclusion is also supported by the results of the retention degree of the wastewater constituents, which increased with membrane operation time (Figure 11). Permeate collection increases the concentration of suspended solids in the feed, which promotes the formation of a filter cake. Furthermore, despite periodic membrane washing, the irreversible fouling increased. Therefore, the results obtained for pristine membranes after the first 2 h of wastewater filtration differed significantly from those obtained at the end of the wastewater UF tests.

**Figure 12:**
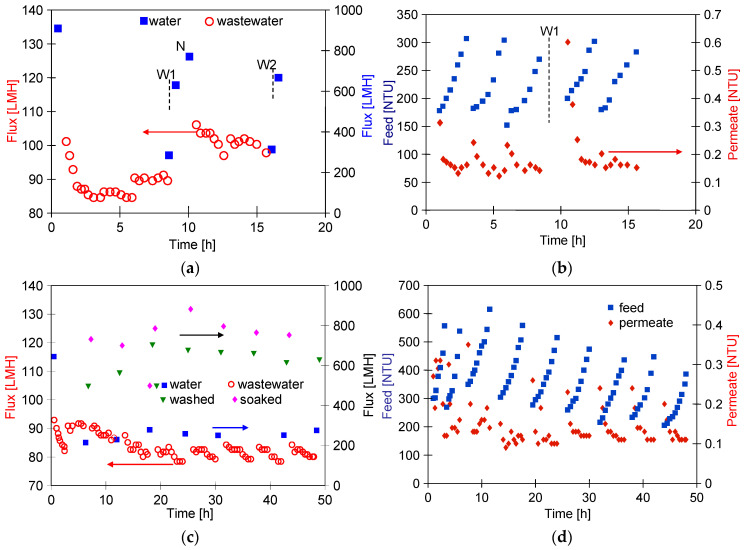
WW2 wastewater separation course using FP200 membranes. (**a**,**b**) Results obtained during 2x concentration of wastewater (VCR = 2) and (**c**,**d**)—2x concentration of retentates obtained (VCR = 4).

The FP200 (200 kDa) membranes have larger pores than FP100 (100 kDa), which may have facilitated internal fouling of the skin layer. As a result, after separation of three portions of wastewater (VCR = 2), the permeate flux decreased to 90 LMH and the pure water flux decreased from 900 to 278 LMH (Figure 12a). After washing with Insect solution and rinsing with DI water, the flux was 624 LMH and increased to 765 LMH after an overnight soak of the membranes in DI water (point N), representing 85% of the initial permeate flux. The significant purification of the membranes by this method is also evidenced by an increase in the permeate turbidity to 0.6 NTU after resumption of WW2 separation (Figure 12b, after 10 h). During the separation of two consecutive samples of wastewater, the permeate turbidity decreased to 0.15–0.2 NTU, and the permeate flux stabilised at 100 LMH.

The smaller decrease in flux indicates that the deposits remaining after washing the membranes (W1) may have contributed to the more porous structure of the reconstituted filter cake. After a further washing of the membranes with Insect solution (W2), the pure water flux was 690 LMH (Figure 12a). In the next step, the retentates obtained in the previous WW2 wastewater filtration (VCR = 2) were separated again reducing their volume by half (total VCR = 4). As a result of a significant increase in the turbidity of the feed (310 NTU), the obtained permeate flux decreased from 100 to 80 LMH (Figure 12c). Systematically repeated washing allowed the flux to be maintained at this level. As before, the UF process was carried out periodically, and the pure water flux determined after a given filtration was at 250 LMH; its value after washing with Insect solution increased to 620 LMH (washed) and after an overnight soak in DI water to 730 LMH (Figure 12c, soaked). Membrane washing caused the turbidity of the permeate to increase to 0.25 NTU, which decreased to 0.1 NTU during UF (Figure 12d). This result confirms that fouling significantly improves separation.

**Figure 13:**
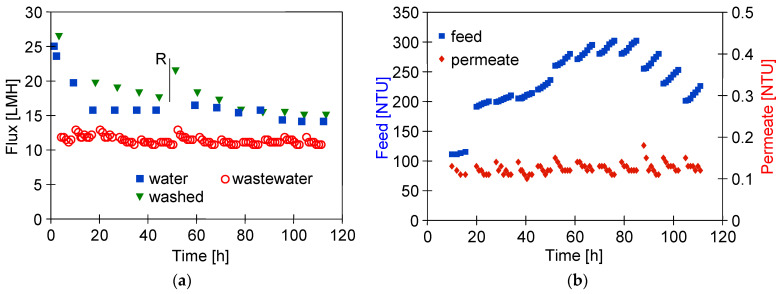
Changes in the permeate flux (**a**) and turbidity (**b**) during separation WW3 wastewater by ESP04 membrane. R—membrane rinsed with the 0.25% sodium disulfite solution for 30 min.

Several works have shown that the fouling intensity can be reduced by using membranes with a low MWCO value [39,40,41]. For this reason, ESP04 membranes, for which the manufacturer declares a MWCO value of 4 kD, were used in the final step. For these membranes, the flux for DI water was at 25 LMH (Figure 13). During wastewater separation, the UF flux decreased to 11 LMH (TMP = 0.1 MPa). Although these membranes had a skin layer much denser than the previously tested membranes, the turbidity of the permeate was 0.1–0.13 NTU (Figure 13b), thus similar to that of the more permeable FP200 membranes (Figure 12d). From this, it can be seen that the separation properties of the resulting filter cake have a significant impact on the degree of separation.

As a result of the fouling, the permeate flux determined for the DI water decreased to 15 LMH. Due to the low flux (compared with FP100 and FP200), the feed (3 L wastewater) was concentrated for nearly 80 h (Figure 13b, 312 NTU). The UF process was carried out 5–6 h/day, after which, the module was rinsed with DI water and washed with Insect solution (30 min). For the first 60 h of testing, this procedure allowed the pure water flux to increase to 20 LMH (Figure 13a, washed). After 50 h of testing, the membranes were preserved for one week by flushing with the 0.25% sodium disulfite (Na_2_S_2_O_5_) solution, which also caused a temporary increase in the permeate flux to 23 LMH. However, finally after 80 h of WW3 separation, the effects of chemical cleaning were already negligible. In the three remaining series, the feed was diluted by adding permeate to it. The resulting reduction in the turbidity of the feed did not change the permeate flux and its turbidity. It also did not improve the effects of the chemical wash. After UF testing, it was found that the surface colour of the ESP04 membranes was still white, in contrast to the FP100 and FP200 membranes, which turned from white to slightly brown after the UF process. This result indicates that it was not the formation of a filter cake, but internal fouling that was the main reason for the changes in performance of the ESP04 membranes.

**Figure 14:**
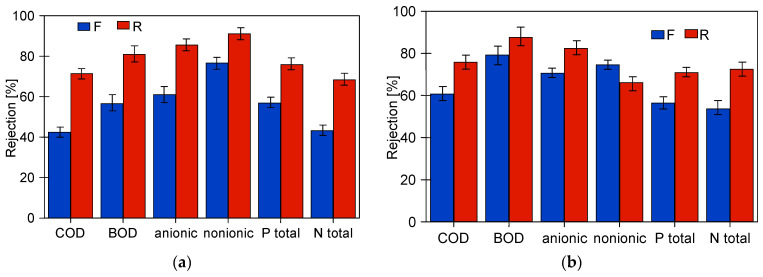
Change in retention rate of wastewater components (**a**) WW2 (FP200) and (**b**) WW3 (ESP04). F—permeate sample taken after the first 2 h of wastewater filtration. R—permeate sample taken at the end of the presented studies (FP200—Figure 12c, ESP04—Figure 13a).

Analysis of the composition of the permeate obtained showed that, as for FP100 (Figure 11), the retention degree increased with the service life of the membranes tested (Figure 14). As expected, the highest rejection values were obtained for ESP04 membranes, which also had the best dextran retention (Figure A1). These membranes also showed the smallest difference in change in degree of separation (values R and F). This is due to the fact that these membranes, having significantly smaller pores in the skin layer (4 kDa), were more resistant to internal fouling than the other membranes (100–200 kDa).

**Figure 15:**
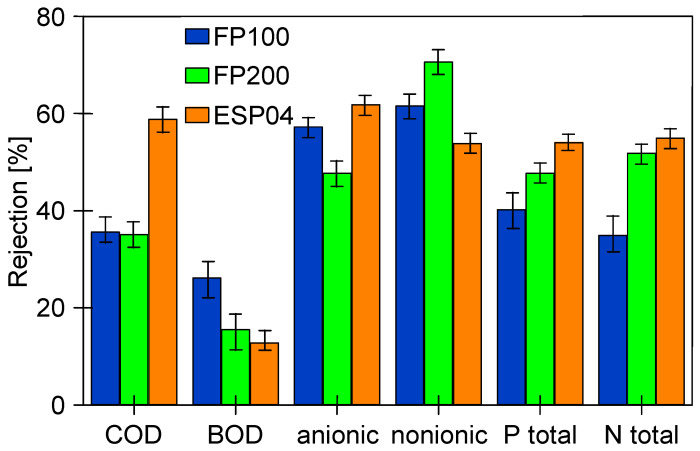
Rejection calculated to the values obtained for the pre-filtered feed (Table 2, —F).

The suspended solids have a significant impact on the results of the parameters tested. Table 2 shows how their values change for the tested wastewater after filtering through the filter paper, which removed most of the suspended solids. As a result, most of the parameters tested decreased, which is due to the fact that components of the wastewater can precipitate or adsorb on the surface of the suspension [3,42]. A comparison of the retention rates calculated in relation to the filtered feed is shown in Figure 15. Although the retention rates have decreased, they are still above 50% for the majority, indicating that dissolved wastewater ingredients were also partially retained by the membranes tested.

### 3.4. Membrane Fouling

**Figure 16:**
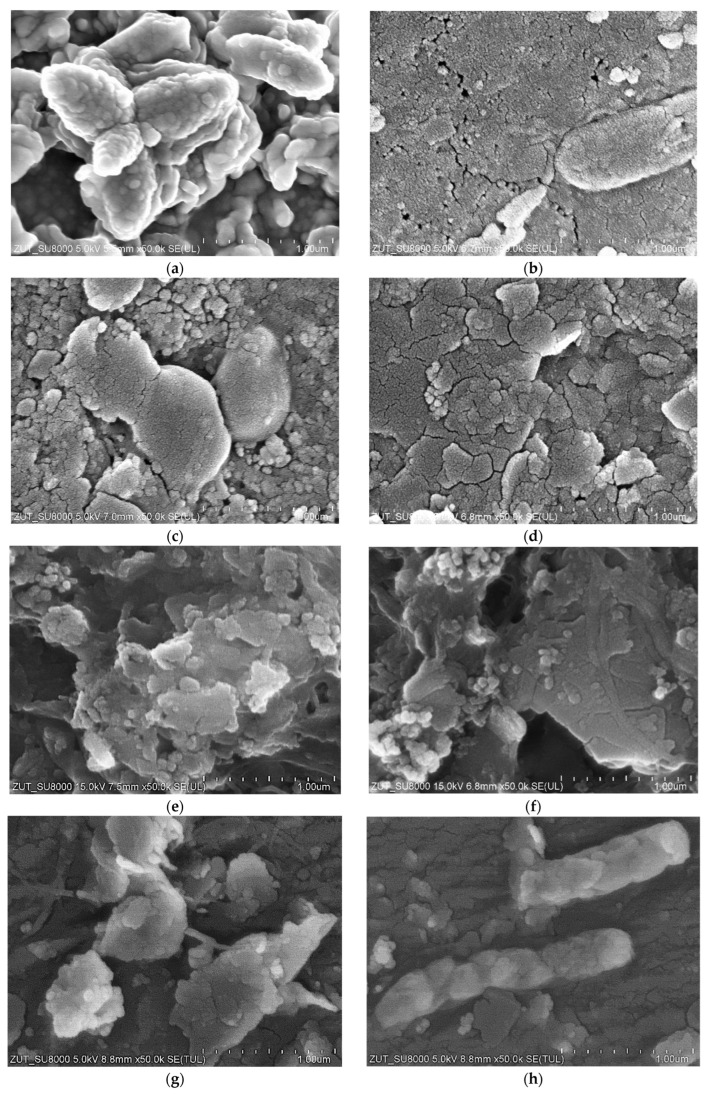
SEM images of studied tubular membrane surfaces after wastewater separation: (**a**) FP100 —WW1, (**c**) FP100—WW2, (**e**) FP200—WW2, and (**g**) ESP04—WW3; and after fouled membrane washing with Insect solution: (**b**) FP100—WW1, (**d**) FP100—WW2, (**f**) FP200—WW2, and (**h**) ESP04—WW3.

SEM observations confirmed that in each of the examined cases, the membrane fouling occurred during the separation of wastewater from the car wash (Figure 16). The filter cakes created were porous; hence, they only partially limited the permeate flux. As expected, the smallest number of deposits formed on the surface of ESP04 membranes (Figure 16g). This result confirms that reducing the MWCO value below 10 kDa makes it possible to reduce fouling [43]. The tested tubular membranes had the same diameter (12.5 mm) and feed flow rate (1 m/s); hence, there was similar flow turbulence. It can therefore be assumed that the differences in the formation of deposits depended on the permeate flux and the resulting polarization phenomena [44]. The structure of the filter cake also depended on the composition of the wastewater. The deposits formed during the filtration of WW2 wastewater created a more compact structure on the membrane surface than those for WW1, which resulted in a greater decrease in the permeate flux (Figure 9). In the deposits formed by WW2, in addition to large agglomerates, numerous particles smaller than 0.1 μm are also visible (Figure 16c), which explains the reason for the slightly higher turbidity of the permeate obtained from WW2 wastewater (Figure 9b). Wastewater from the car wash may have different components [3,20]; hence, the resulting filter cake differed not only in structure but also in the composition (Table 3). Significantly more pollutants were found in WW2 wastewater. Structure and composition also influence the efficiency of wastewater separation. WW2 contained much larger amounts of Si, the compounds of which cause the formation of deposits that are difficult to remove (Figure 16d). Smaller amounts of Si were also detected on the surfaces of the remaining membranes, which, given the significant amount of oxygen detected, indicates SiO_2_; its presence can improve the separation properties [45], which may be one of the reasons for the increase in the degree of separation of wastewater components with the time of membrane operation (Figure 11 and Figure 14). The presence of bacterial cells was also found in the examined sediments. In previous works, nearly 40 types of bacteria were detected in car wash wastewater, including many antibiotic-resistant ones [46]. Single bacterial cells were also observed in membrane samples after washing them with Insect solution. Examples of rod-shaped bacteria can be seen in Figure 16b,h.

## 4. Conclusions

Results obtained in the present study demonstrated that the tubular membranes can be successfully used for the separation of high-turbidity car wash wastewater. The 2–3 times increase in the feed turbidity resulting from permeate collection only slightly reduced the obtained permeate flux.

During the UF process of wastewater, intense fouling occurs, which causes a rapid decrease in the permeate flux. The use of membrane washing (every 5–7 h) with 0.5% Insect solution (30 min) allowed for the reduction of the intensity of fouling and for the stabilisation of the flux at a favourably high level.

Depending on the wastewater composition and the membranes used, the permeate flux in the range of 50–100 LMH was obtained, which is below 20% of the initial permeate flux. For membranes with the smallest pores (MWCO = 4 kDa), the fouling intensity was lower, and the permeate flux was at the level of 15 LHM (60% initial flux).

It has been documented that the deposit layer formed on the surface of the membranes has a significant impact on the separation degree. As a result, a permeate of similar quality was obtained for the FP200 membranes (200 kDa) as was for the ESP04 membranes (4 kDa), with 5 times higher efficiency (80 LMH).

## Figures and Tables

**Table 1:** Parameters of applied PCI tubular membranes [29].

Parameter	FP100	FP200	ESP04
material	PVDF	PVDF	Modified PES
MWCO	100 kDa	200 kDa	4 kDa
diameter [mm]	12.5	12.5	12.5
pH range	1.5–12	1.5–12	1–14
max TMP [MPa]	1	1	3
max T [K]	353	353	338

**Table 2:** Parameters of wastewaters (WW) collected from the touchless car washes (1–3).

Parameter	WW 1	WW1-F ^1^	WW 2	WW2-F ^1^	WW 3	WW3-F ^1^
COD [mg/L]	1050	800	962	695	1120	680
BOD [mg/L]	370 a	324	195	38	260	75
anionic [mg/L]	135	133	99.2	56	106	71
nonionic [mg/L]	15.3	6.9	10.4	4.1	13.5	6.1
total P [mg/L]	14.5	12.9	7.6	7.4	13.8	12.1
total N [mg/L]	12.6	7.2	7.5	5.1	17.8	7.3
TSS [mg/L]	365	-	220	-	242	-
turbidity [NTU]	155	42	190	56	179	60

^1^ wastewater after pre-filtration (paper filter).

**Table 3:** Results of SEM–EDX analysis of deposits formed on the FP100 and ESP04 membrane surfaces. 1, 2, 3—wastewater WW1, WW2, and WW3. W—washed membrane (0.5% Insect solution). The elemental content is presented in percentages [wt%].

Element	FP100-1	FP100-1W	FP100-2	FP100-2W	ESP04-3	ESP04-3W
C	29.60	33.19	28.72	22.78	50.26	50.24
F	60.61	66.45	10.32	47.24	-	-
O	9.0	-	45.15	23.69	42.11	42.71
Fe	0.34	-	0.92	0.53	0.08	-
Al	0.24	0.21	2.91	1.81	0.24	0.03
Si	0.21	0.15	6.15	2.85	0.56	0.21
Mg	-	-	0.52	0.37	-	-
P	-	-	0.99	-		-
S	-	-	2.25	0.55	6.75	6.81
K	-	-	0.26	0.18		-
Ca	-	-	0.87	-		-
Zn	-	-	0.94	-		-

## Data Availability

Raw data generated during the study was deposited: Gryta, M. & Woźniak, P. (2024). Tubular ultrafiltration car wash wastewater (1–) [dataset]. https://mostwiedzy.pl/pl/open-research-data/tubular-ultrafiltration-car-wash-wastewater,626013059659940-0.

## Appendix A

**Table A1:** Parameters of feed and permeate.

Parameter [mg/L]	Feed	Feed-F ^1^	Permeate	Retentate	Retentate-F ^1^	Permeate R^2^
FP100-WW1						
COD	1026	800	724	2257	1508	721
BOD	368	344	103	362	345	112
anionic	130	133	72	305	260	81
P total	14.4	12.8	6.6	32.1	27.5	7.5
N total	12.5	7.2	7.5	27.4	17.8	6.7
FP200-WW2						
COD	998	637	515	1644	816	525
BOD	68	15	12	79	18	12
anionic	92.5	56	33.2	264	89	38
nonionic	10.4	4.1	2.4	31.2	8.8	3.4
P total	7.6	7.4	5.2	14.2	10.2	6.1
N total	7.6	5.1	4.8	14.4	10.7	7.0
ESP04-WW2						
COD	775	689	304	1346	788	324
BOD	266	60	55	486	65	60
anionic	106	70.6	31	177	81.7	31.2
nonionic	13.5	6.4	3.4	16.8	12.3	5.7
P total	12.8	11.3	5.6	21.7	13.8	6.3
N total	8.5	6.4	3.9	16.6	10.1	4.6

^1^ wastewater after filtration; ^2^ Permeate R—retentate as a feed.

## Appendix B

Dextran separation.
